# Dataset of noise signals generated by smart attackers for disrupting state of health and state of charge estimations of battery energy storage systems

**DOI:** 10.1016/j.dib.2024.111200

**Published:** 2024-12-05

**Authors:** Alaa Selim, Huadong Mo, Hemanshu Pota

**Affiliations:** a School of Engineering and Technology, University of New South Wales, Canberra, Australia; b School of Systems and Computing, University of New South Wales, Canberra, Australia

**Keywords:** Noise signals, Battery energy storage systems, State of health estimation, State of charge estimation, Cyber-physical system, Deep reinforcement learning, False data injection attack

## Abstract

This dataset is generated from real-time simulations conducted in MATLAB/Simscape, focusing on the impact of smart noise signals on battery energy storage systems (BESS). Using Deep Reinforcement Learning (DRL) agent known as Proximal Policy Optimization (PPO), noise signals in the form of subtle millivolt and milliampere variations are strategically created to represent realistic cases of False Data Injection Attacks (FDIA). These signals are designed to disrupt the State of Charge (SoC) and State of Health (SoH) estimation blocks within Unscented Kalman Filters (UKF). The low-magnitude noise signals are specifically crafted to be stealthy, evading easy detection while still effectively causing malfunctions in the estimation processes. Additionally, we introduce a verification case using a different battery model and estimation algorithm to enhance generalization. This case involves high-noise signals with defined thresholds for current and voltage noise levels, which cause significant disruptions to Kalman Filters. These signals serve as a complementary example of adversarial attacks, demonstrating how such noise can destabilize estimation algorithms and lead to critical control errors. This dataset is valuable for researchers and engineers aiming to understand and mitigate the effects of smart cyber-physical attacks on BESS. These attacks can disrupt real-time BESS controllers by injecting false input data related to SoC and SoH, leading to physical control manipulations, increased energy costs, inefficiencies in demand-side management, and incorrect day-ahead scheduling, thereby destabilizing grid operations. The data is reusable in studies focused on enhancing the resilience of SoC and SoH estimation methods, as well as in developing robust defensive strategies against smart DRL-based adversarial attacks.

Specifications Table

**Table:** 

Subject	*Electrical and Electronic Engineering*
Specific subject area	Cyber-physical security in battery energy storage systems with DRL-generated stealthy noise attacks on SoH and SoC estimations
Type of data	MATLAB files (.mat file). Figures extracted from the signal analyser tool in MATLAB
Data collection	The data were collected using real-time simulations in MATLAB/Simscape (R2024a and R2024b). Deep Reinforcement Learning (DRL) agent known as Proximal Policy Optimization (PPO), was implemented to generate noise signals. These simulations focused on disrupting State of Charge (SoC) and State of Health (SoH) estimations using Unscented Kalman Filters (UKF) in Battery Energy Storage Systems (BESS). The dataset includes only those scenarios where noise signals successfully impacted the estimation accuracy.
Data source location	Organization: University of New South Wales City: Canberra, ACT Country : Australia
Data accessibility	Repository name: Mendeley Data Data identification number: doi: 10.17632/f7xyzyrc58.1 Direct URL to data: https://data.mendeley.com/datasets/f7xyzyrc58/1 To begin your experiments, download one of the datasets provided—Datamodel_with_noises_Case1.mat or Datamodel_with_noises_Case2.mat—from the available files. Please note: These two datasets represent different scenarios, so only one should be loaded into the MATLAB workspace at a time. Case 1: Represents a scenario with high noise values, simulating a more aggressive attack on the battery system. Case 2: Represents a scenario with very low noise values, intended to simulate a stealthier, harder-to-detect attack. Validation case: Represents a scenario with high noise levels, that were able to achieve high error in estimation algorithms for different battery model and SoC estimation algorithm. Once you have downloaded the desired dataset, load it into the MATLAB workspace. After the dataset is loaded, you can proceed to run your simulations and experiments following the procedure in https://data.mendeley.com/datasets/f7xyzyrc58/1.
Related research article	None

## 1 Value of the Data

• The data provide detailed noise signals specifically crafted to interfere with SoC and SoH estimations in BESS. These noise signals are generated using a DRL algorithm and are finely tuned to operate within the millivolt and milliampere range, making them difficult to detect but still effective in causing disruptions. This dataset is valuable for researchers aiming to explore the impact of such noises on BESS.
• Researchers can reuse these data to test and improve the robustness of their SoC and SoH estimation algorithms against stealthy noise attacks. The data serve as a challenging benchmark for developing more resilient estimation methods that can maintain accuracy even when faced with low-level, sophisticated noise.
• The dataset is generated using real-time simulations in MATLAB/Simscape, making it highly applicable for researchers looking to validate their models or algorithms under realistic conditions. This ensures that findings derived from this dataset are relevant to real-world applications in BESS.
• The data can be employed to study the effectiveness of various defense mechanisms against DRL-generated noise attacks. Researchers can use this dataset to experiment with and evaluate different strategies for detecting and mitigating stealthy attacks in BESS.
• Given its focus on subtle and difficult-to-detect noise, this dataset is particularly useful for developing and testing advanced cyber-physical security measures. It provides a foundation for studying how small perturbations in current and voltage measurements can lead to significant disruptions, helping to improve the security and reliability of BESS*.*

## 2 Background

The motivation for compiling this dataset is driven by the urgent need to understand and mitigate the vulnerabilities of battery energy storage systems to cyber-physical attacks, particularly False Data Injection Attacks (FDIA). FDIA can compromise sensor measurements, such as voltage and current, which are essential for accurate SoC and SoH estimations. In real-world scenarios, even minor inaccuracies in these measurements can lead to significant issues, such as incorrect battery charging or discharging cycles, reduced battery lifespan, or even system-wide failures in energy management. For instance, in grid-connected BESS, inaccurate SoC readings could result in improper power dispatch decisions, while compromised SoH data could lead to undetected battery degradation, increasing the risk of unexpected outages. The dataset provides a detailed collection of noise signals specifically designed to exploit these vulnerabilities, simulating realistic FDIA scenarios. By understanding how these subtle, undetectable attacks can disrupt critical battery management functions, researchers can better develop and test robust defense mechanisms to enhance the security and reliability of BESS in modern power grids.

## 3 Data Description

**Fig. 1:**
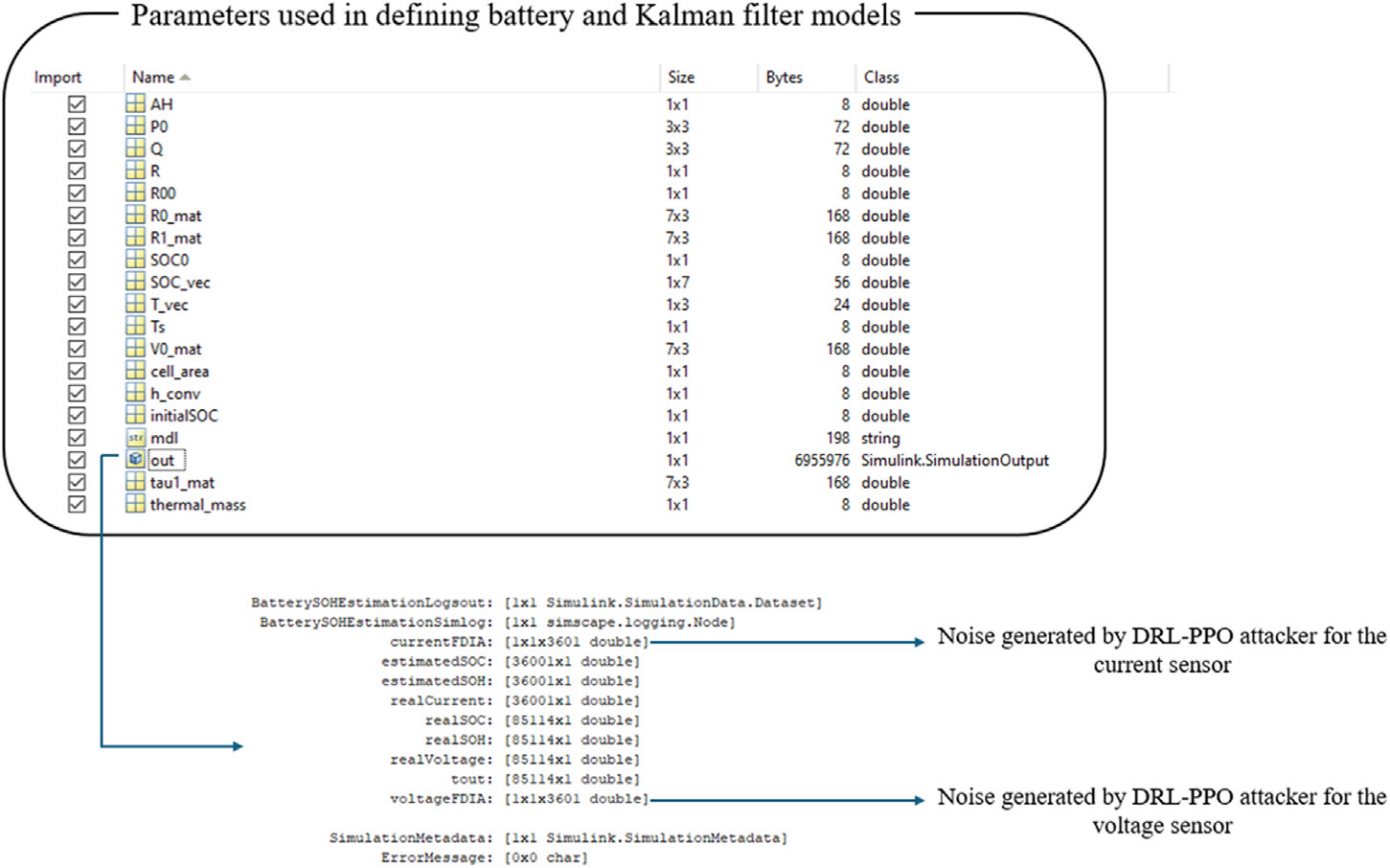
Dataset of noise signals and model parameters to reproduce this experiment.

The provided data are in .mat format, suitable for use in MATLAB. This dataset includes both battery parameters and Kalman filter parameters used in the study as shown in Fig. 1. Specifically, the battery parameters define the characteristics and behavior of the battery model, while the Kalman filter parameters are used for SoC and SoH estimations.

Within the dataset, the out structure contains noise values for current (currentFDIA) and voltage (voltageFDIA) measurements. These noise values have been generated by DRL-PPO model, designed to simulate realistic attack scenarios on BESS.

Two cases are provided:

• **Case 1:** High noise values, simulating a more aggressive attack scenario.
• **Case 2:** Very low noise values, representing a stealthier, harder-to-detect attack.

Researchers can load this data into the MATLAB workspace and simulate the impact of these noise signals on BESS. The noise signals are intended to disrupt the SoC and SoH estimation processes, providing a realistic scenario for studying FDIA in BESS.

Additional case for concept verification:

This verification case focuses on identifying and analyzing the threshold at which adversarial noise signals cause massive disruptions to SoC estimation processes. By systematically tuning the noise signal parameters, we aim to determine the minimum signal characteristics—such as amplitude and frequency—that can lead to significant deviations in the estimated SoC values. The goal is to establish a clear threshold for disruption that adversarial attacks could exploit while maintaining a balance between stealth and impact. This case provides critical insights into the vulnerabilities of estimation processes under adversarial conditions and helps define the limits of system resilience.

## 4 Experimental Design, Materials and Methods

The data were acquired through a series of real-time simulations conducted in MATLAB/Simscape (version R2024a) [1]. Additionally, the example was validated through real-time testing on a Speedgoat Performance real-time target machine equipped with an Intel® 3.5 GHz i7 multi-core CPU. The model was capable of running in real time with a step size of 100 µs, ensuring the robustness and applicability of our approach in practical scenarios. The primary objective of these simulations was to generate and analyze noise signals that could stealthily disrupt SoC and SoH estimations within BESS to emulate a scenario of FDIA [[2], [3], [4], [5]]. The process involved several key components and steps, which are detailed below:

### 4.1 Simulation environment

**Fig. 2:**
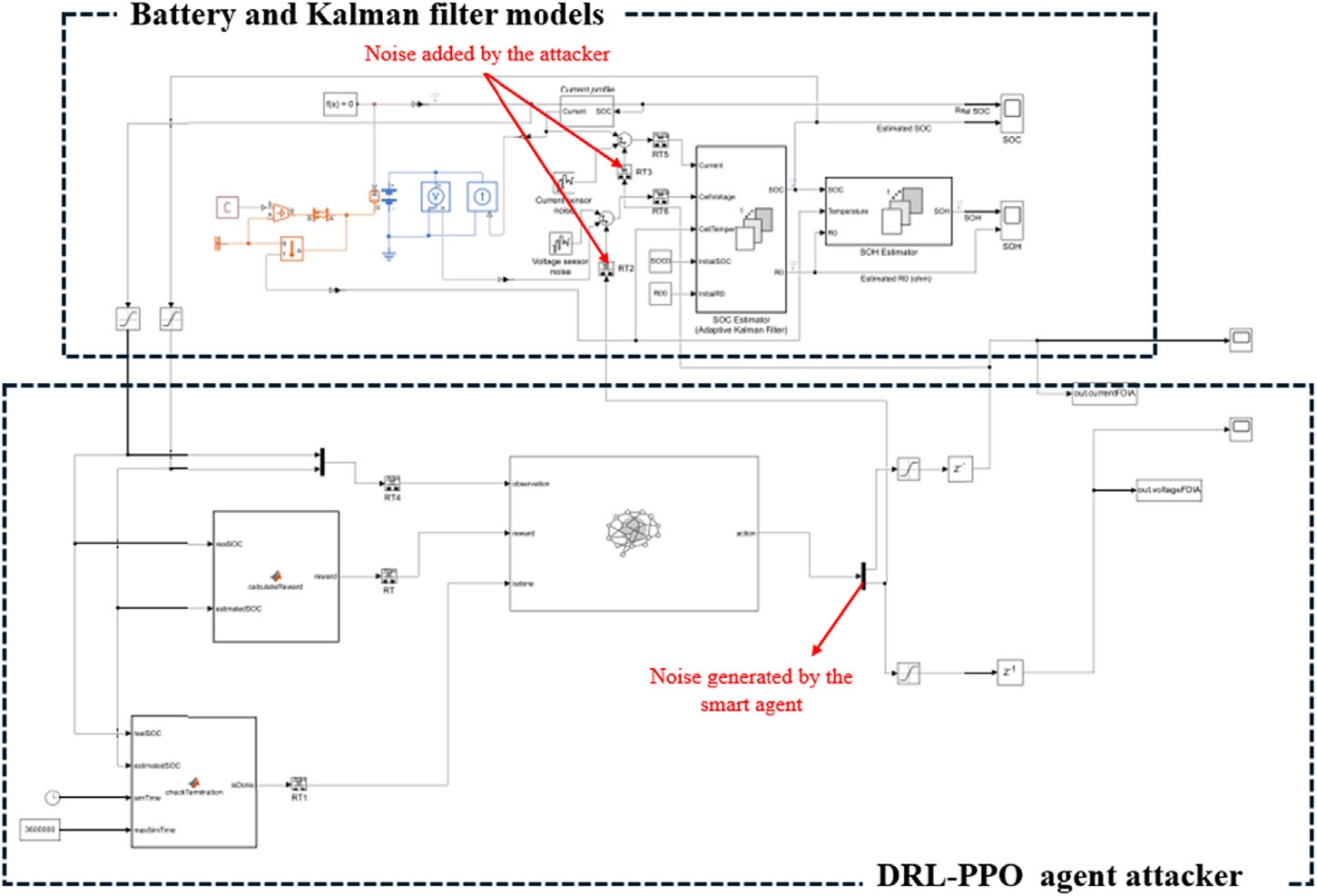
Experimental test setup.

Software: MATLAB/Simscape (R2024a) was used to create a realistic simulation environment replicating BESS. The system modeled included key components such as the battery, charging/discharging mechanisms, and UKF for SoC and SoH estimation as shown in Fig. 2.

Battery Model: A Li-ion battery model was employed within Simscape, incorporating parameters such as capacity, voltage, and internal resistance, to mimic real-world battery behavior. (its model available at: https://www.mathworks.com/help/simscape-battery/ug/battery-state-of-health-estimation.html)

### 4.2 Verification case

**Fig. 3:**
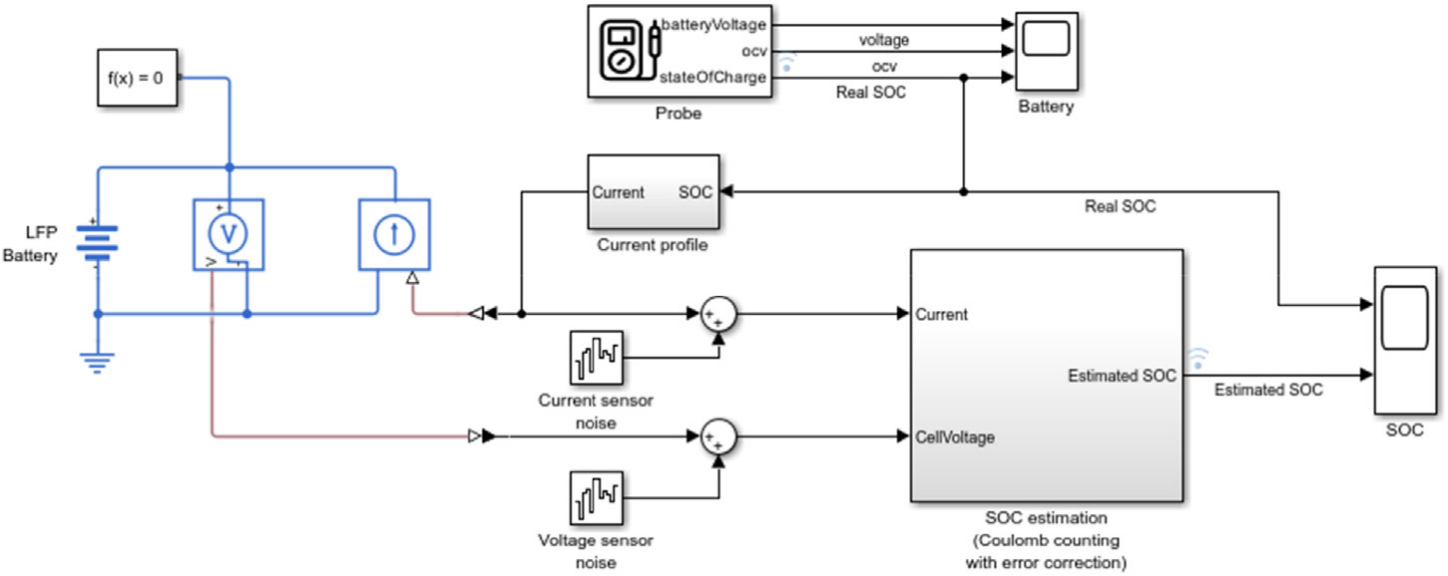
Experimental test setup for the verification case.

In this additional case, we explore the impact of high-noise adversarial signals on the SoC estimation process using MATLAB 2024b (https://www.mathworks.com/help/simscape-battery/ug/estimate-soc-of-lithium-iron-phosphate-battery.html) simulations with Lithium Iron Phosphate (LiFePO4) battery models (see Fig. 3). The Extended Kalman Filter (EKF) is employed for SoC estimation, offering a robust framework for handling non-linear dynamics inherent in battery systems. The high-noise signals are strategically designed to exhibit significant variations while maintaining a low Signal-to-Noise Ratio (SNR), disrupting estimation processes. Although these signals are more easily detected due to their magnitude, their ability to significantly degrade EKF performance highlights the critical vulnerabilities of estimation algorithms to adversarial attacks. The case demonstrates how high-noise signals can corrupt the filter's predictive and corrective mechanisms, resulting in SoC deviations that could propagate downstream control processes, leading to operational inefficiencies.

### 4.3 Noise signal generation

Algorithm: DRL-PPO agent [6] was utilized to generate the noise signals. PPO was chosen for its balance between exploration and exploitation, which is critical for creating effective yet subtle noise patterns.

Training Process: The DRL model was trained to introduce perturbations in the form of voltage (millivolts) and current (milliamperes) fluctuations that could interfere with the UKF estimation processes [7]. The training process involved multiple iterations, with the model continuously adjusting to minimize detection while maximizing disruption. The agent's state space includes voltage, current, and SoC/SoH estimations, while the action space involves introducing subtle noise signals in the millivolt and milliampere range. The reward function is designed to maximize disruption of the SoC and SoH estimations while minimizing noise injection. The agent trains over multiple episodes using MATLAB/Simscape, with the training parameters (as shown in Table 1), carefully chosen to balance exploration and exploitation. The dataset focuses on the generated noise signals, while further details about the DRL model and training process will be shared in a subsequent publication for deeper understanding.

**Table 1:** DRL-PPO training parameters.

Parameter	Value
Max Episodes	120
Max Episode Length	3600
Stopping Criteria	None
Average Window Length	10
Sample Time	10
Discount Factor	0.99
Execution Environment	GPU
Batch Size	128
Experience Horizon	512
Entropy Loss Weight	0.01
Actor Optimizer Learn Rate	0.001
Actor Optimizer Gradient Threshold	Inf
Critic Optimizer Learn Rate	0.001
Critic Optimizer Gradient Threshold	Inf

### 4.4 Data collection

**Fig. 4:**
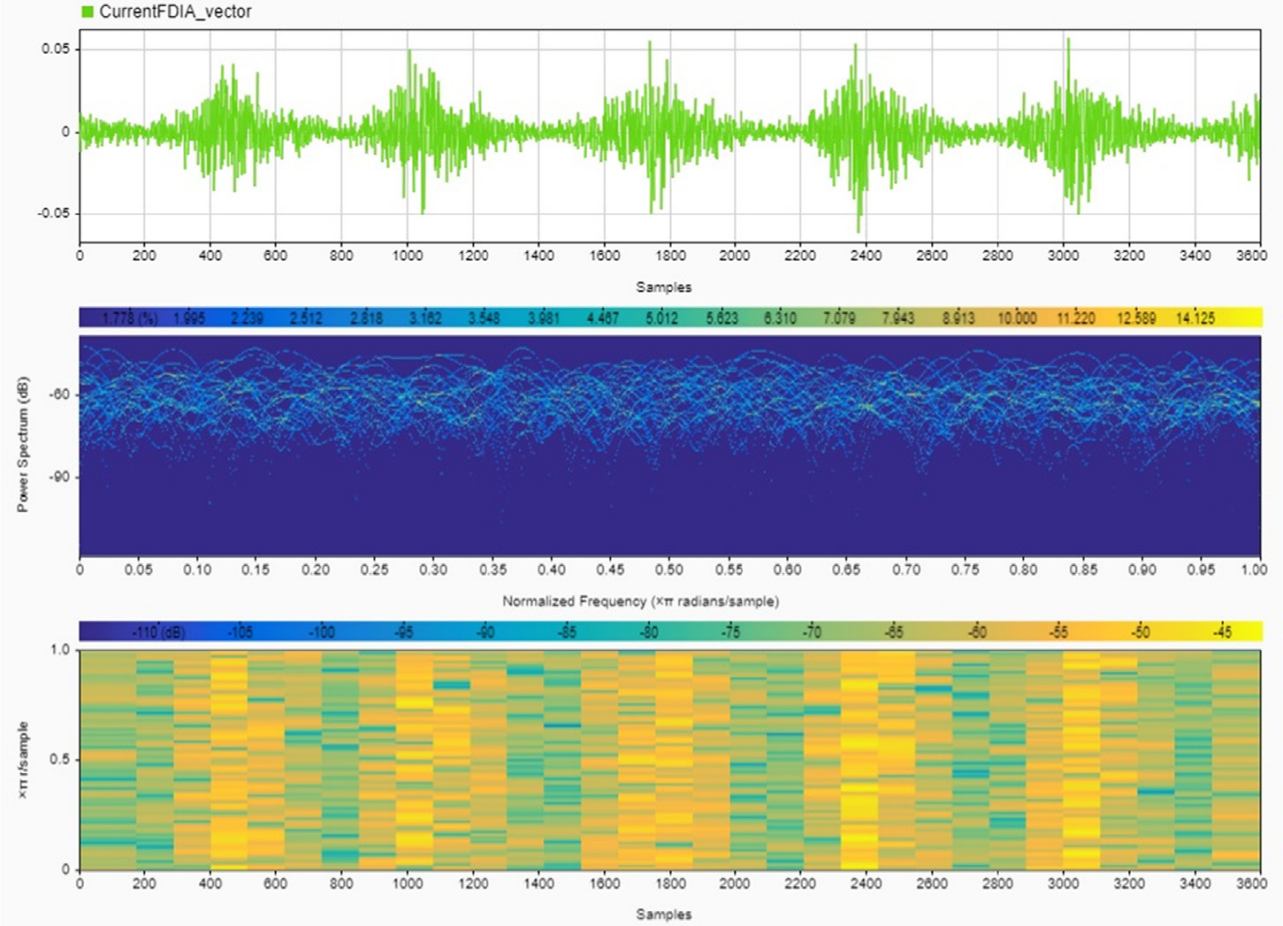
Current noise signals due to the FDIA analyzed using a signal analyzer tool in MATLAB to show its magnitude in the upper plot, power spectrum in the middle plot and normalized frequency in the bottom plot – Case 1.

**Fig. 5:**
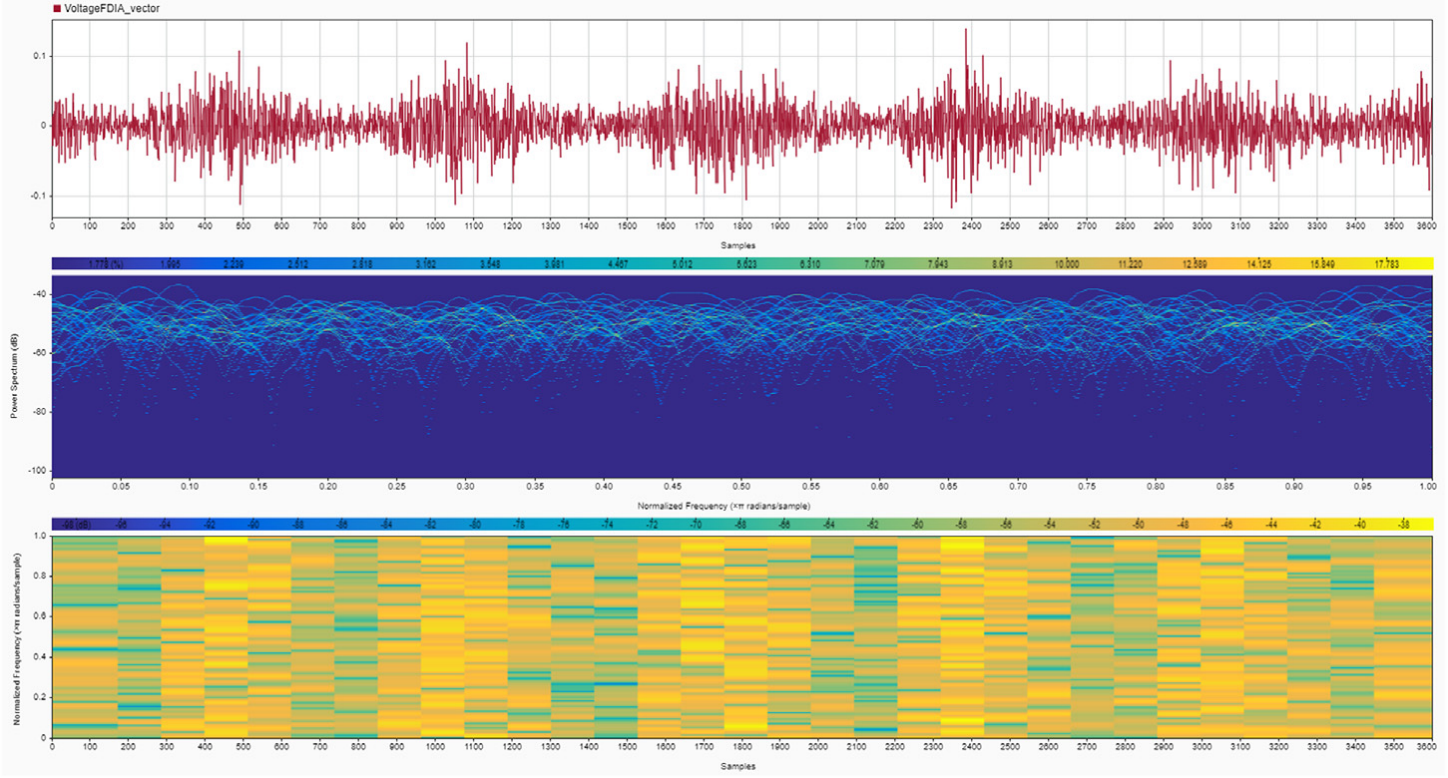
Voltage noise signals due to the FDIA analyzed using a signal analyzer tool in MATLAB to show its magnitude in the upper plot, power spectrum in the middle plot and normalized frequency in the bottom plot – Case 1.
• **Case 2:** Very low noise values, representing a stealthier, harder-to-detect attack (shown in Fig. 6, Fig. 7).

Instruments: The noise signals and their effects on SoC and SoH estimates were recorded using MATLAB's built-in data logging functions [8]. The recorded data include the time series of voltage, current, SoC, SoH, and the corresponding noise signals [9]. Two cases are provided for the noise signals:

• **Case 1:** High noise values, simulating a more aggressive attack scenario (shown in Fig. 4, Fig. 5).

**Fig. 6:**
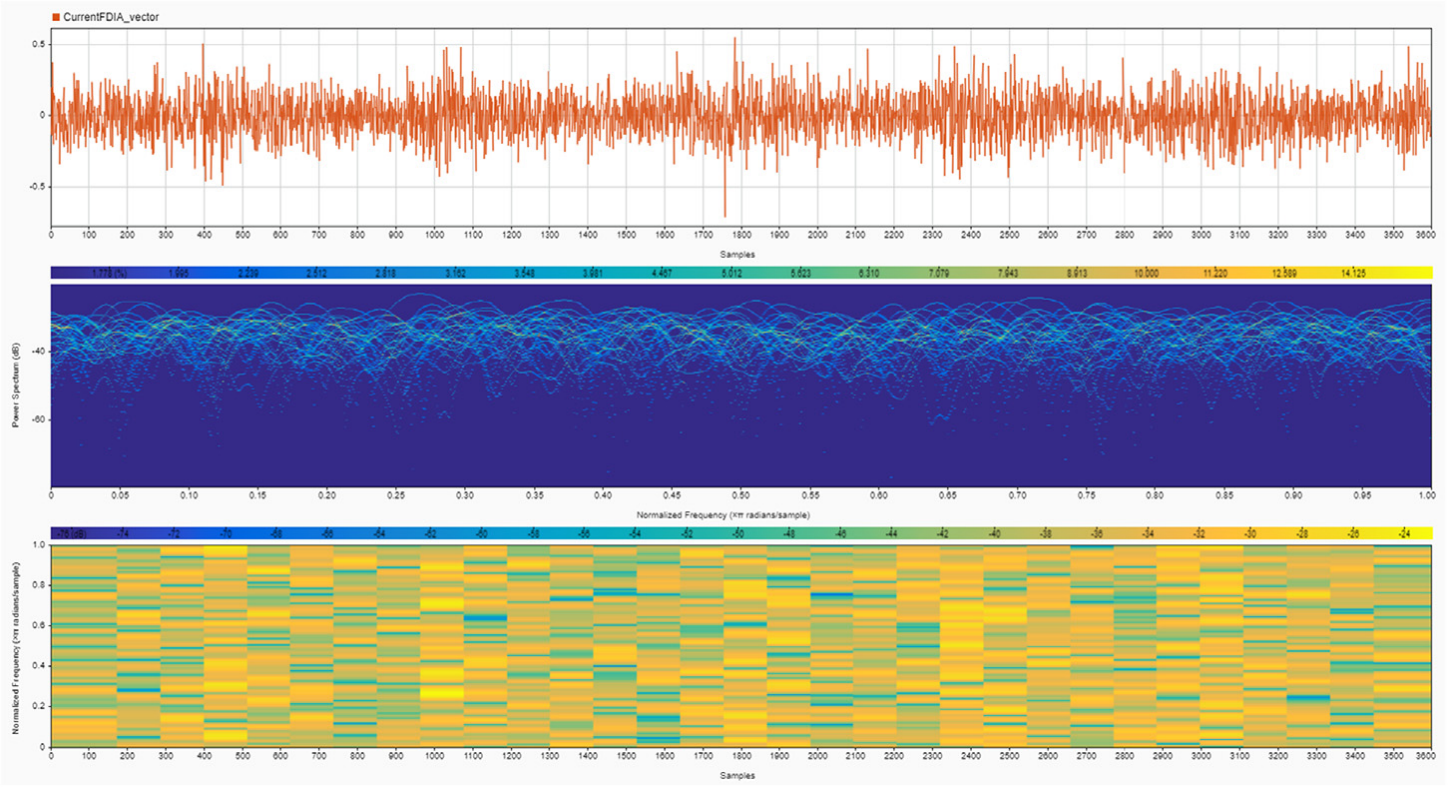
Current noise signals due to the FDIA analyzed using a signal analyzer tool in MATLAB to show its magnitude in the upper plot, power spectrum in the middle plot and normalized frequency in the bottom plot – Case 2.

**Fig. 7:**
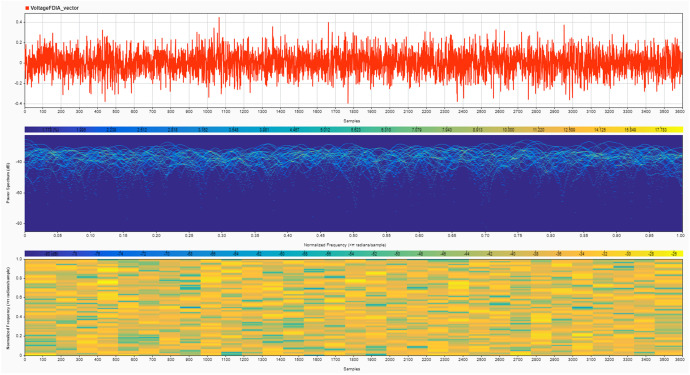
Voltage noise signals due to the FDIA analyzed using a signal analyzer tool in MATLAB to show its magnitude in the upper plot, power spectrum in the middle plot and normalized frequency in the bottom plot – Case 2.

While the high noise value scenario simulates more aggressive attacks, it is important to clarify that these noise levels remain within the permissible ranges defined by international standards, such as IEC 61,000–4-7 [10] and IEEE 1159 [11]. This adherence to standard noise thresholds makes even these aggressive attacks harder to detect, as they effectively mimic the typical operational fluctuations observed in battery systems. Additionally, the high noise scenario may include stealthy spikes designed to disrupt state estimators while still avoiding detection by staying within the acceptable noise levels for normal system operations.

Conditions: the noise signals were tested extensively under various battery states, including different levels of charge and discharge cycles, to ensure their significant impact on SoC and SoH estimation. The results, as shown in the provided .mat files, demonstrate a substantial disruption in the SoC and SoH estimation processes, confirming the powerful influence of these noise signals on the BESS.

**Fig. 8:**
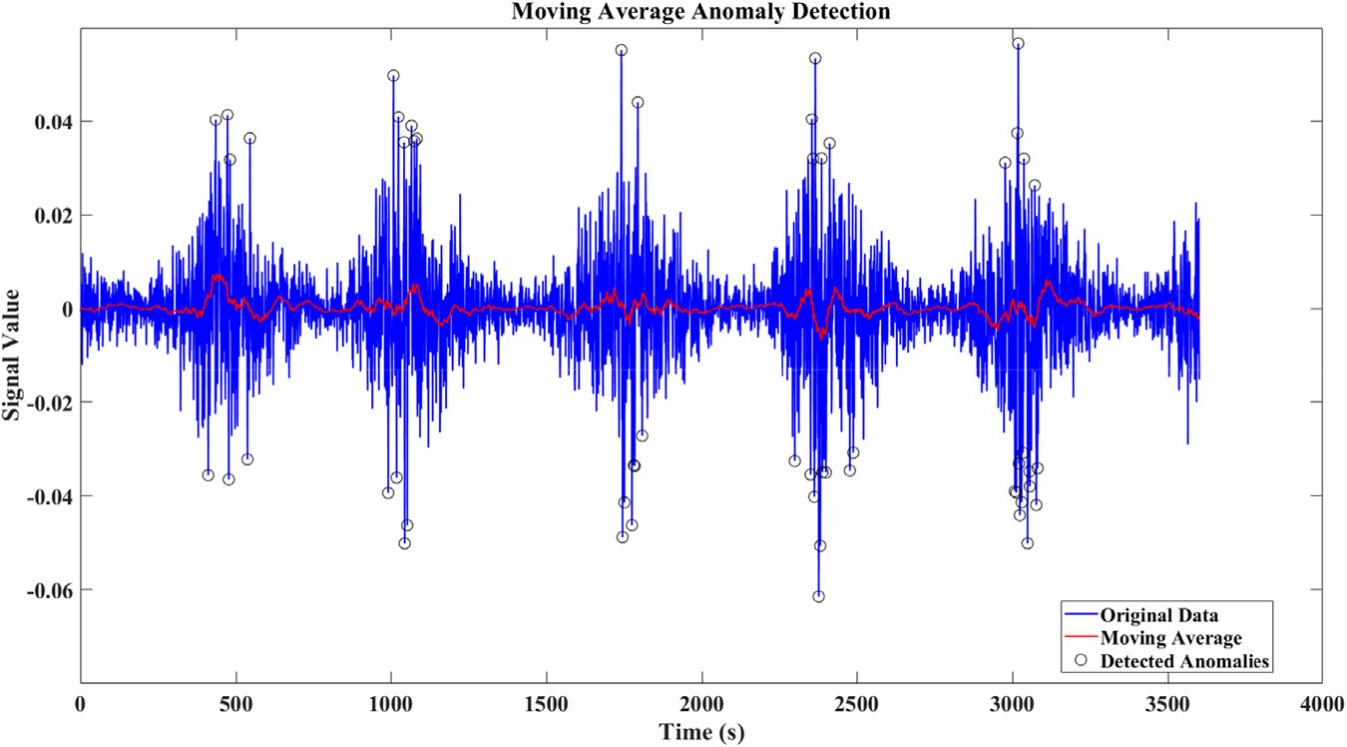
Moving average anomaly detection for current noise signal-Case 1.

**Fig. 9:**
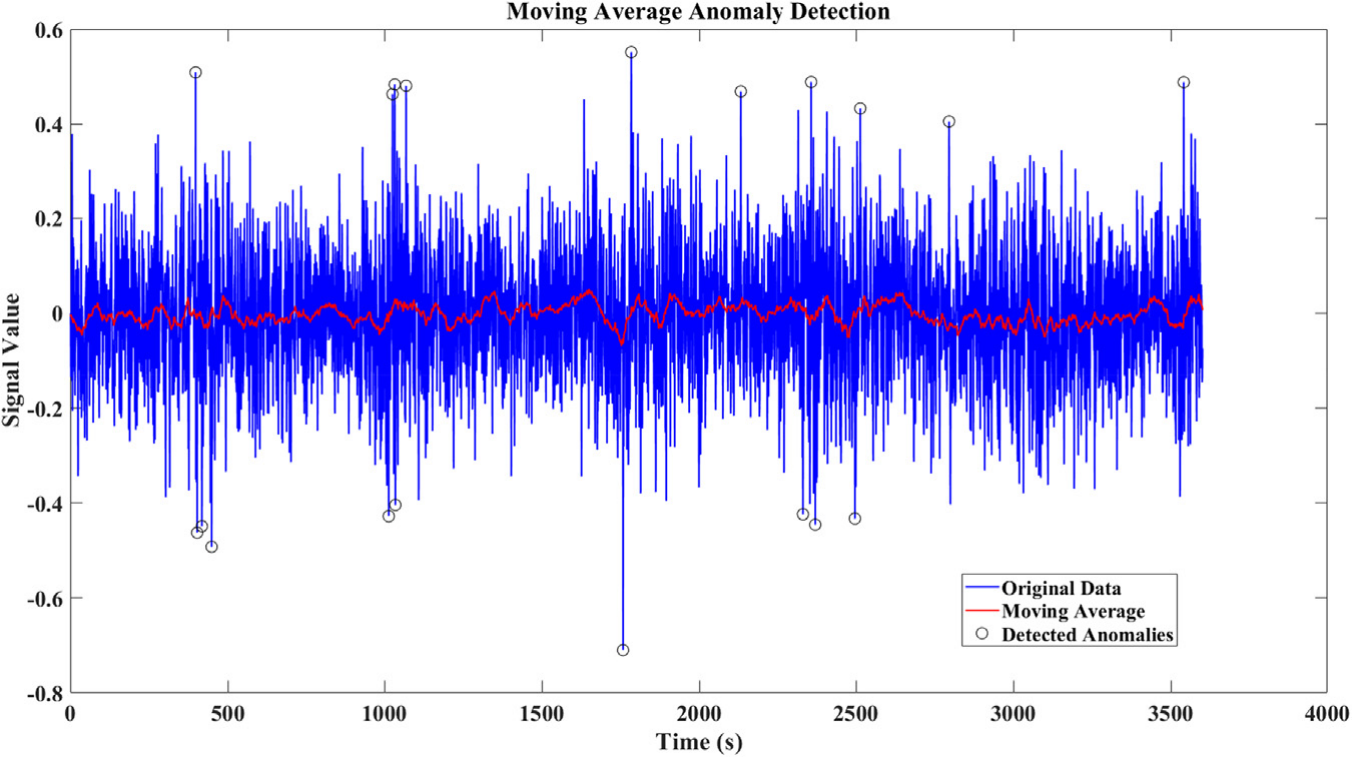
Moving average anomaly detection for current noise signal-Case 2.

In this work, we employed a moving average method to monitor potential anomalies in the measurement data. This method helps smooth short-term fluctuations and highlight trends in the residuals of variables such as SoC and SoH. The detection threshold was calibrated based on noise levels defined by standards such as IEC 61,000–4-7 and IEEE 1159, ensuring that only significant deviations from normal operational noise trigger detection. As shown in Fig. 8, Fig. 9, the anomalies introduced by FDIA remain subtle and are difficult to distinguish from normal system noise, as they stay within the permissible range of millivolt and milliampere fluctuations. This highlights the stealthy nature of these attacks and the challenge of detecting them with traditional methods.

**Fig. 10:**
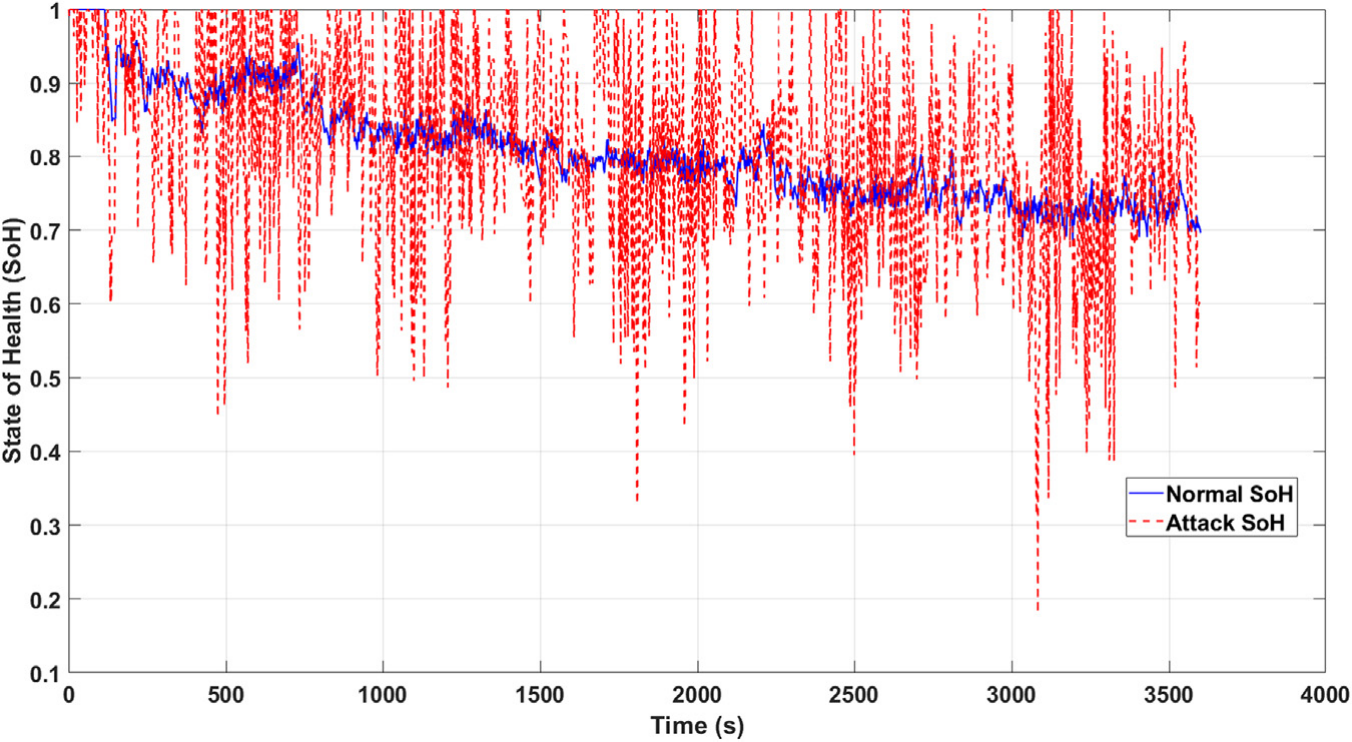
SoH estimation impacted by noise signals caused by attacks.

Fig. 10 illustrates how the smart noise signals generated by the DRL agent affect the SoH estimators, which represent the final stage of the model. The stealthy noise introduces subtle perturbations that disrupt the accuracy of the SoH estimation, leading to deviations from the true SoH values. These oscillatory deviations can result in incorrect battery management decisions, such as improper charging or discharging, potentially causing long-term physical degradation of the battery system.

### 4.5 Verification case

**Fig. 11:**
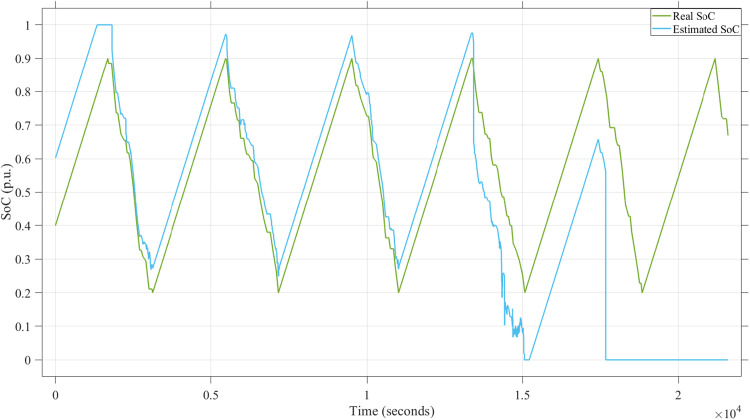
SoC estimation impacted by noise signals caused by attacks.

Fig. 11 illustrates the performance of the SoC estimation process by comparing the real SoC (green line) and the estimated SoC (blue line) over time. It shows the impact of the adversarial noise signals on the estimation accuracy. While the real and estimated SoC values initially align closely, significant deviations occur after *t* = 1.5 × 10^4 s, with the estimated SoC diverging drastically from the actual value. This indicates that the adversarial noise signals effectively disrupt the Kalman Filterʼs ability to maintain accurate SoC estimation, potentially leading to critical errors in system operation.

**Fig. 12:**
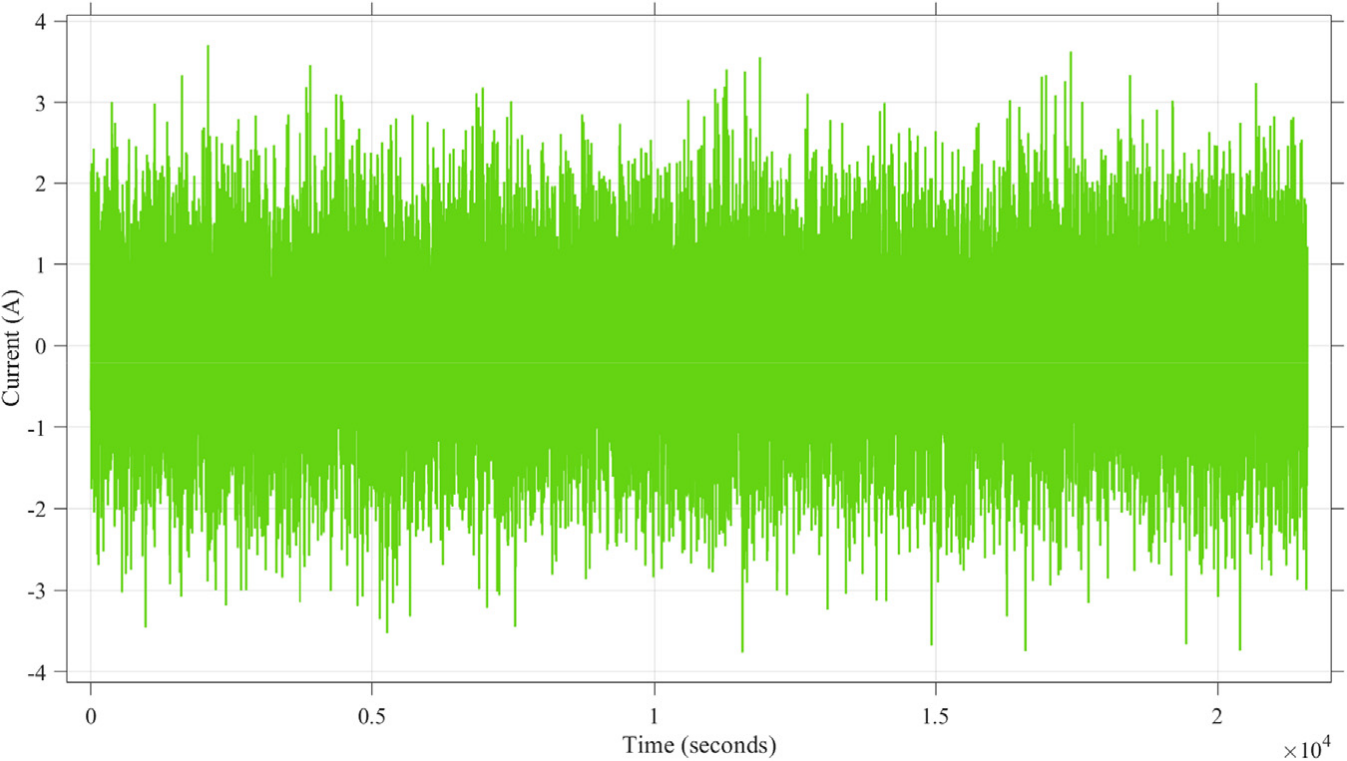
Current noise attack using Band-Limited White Noise block noise power of 1.

Fig. 12 presents the current profile used in the simulation, which includes a noise signal component with an amplitude range of approximately −3 A to +3 A. The random fluctuations in the current demonstrate the adversarial noiseʼs influence on the input parameters, which directly affects the accuracy of SoC estimation. The variability in this current profile highlights the dynamic nature of the disturbance introduced into the system.

**Fig. 13:**
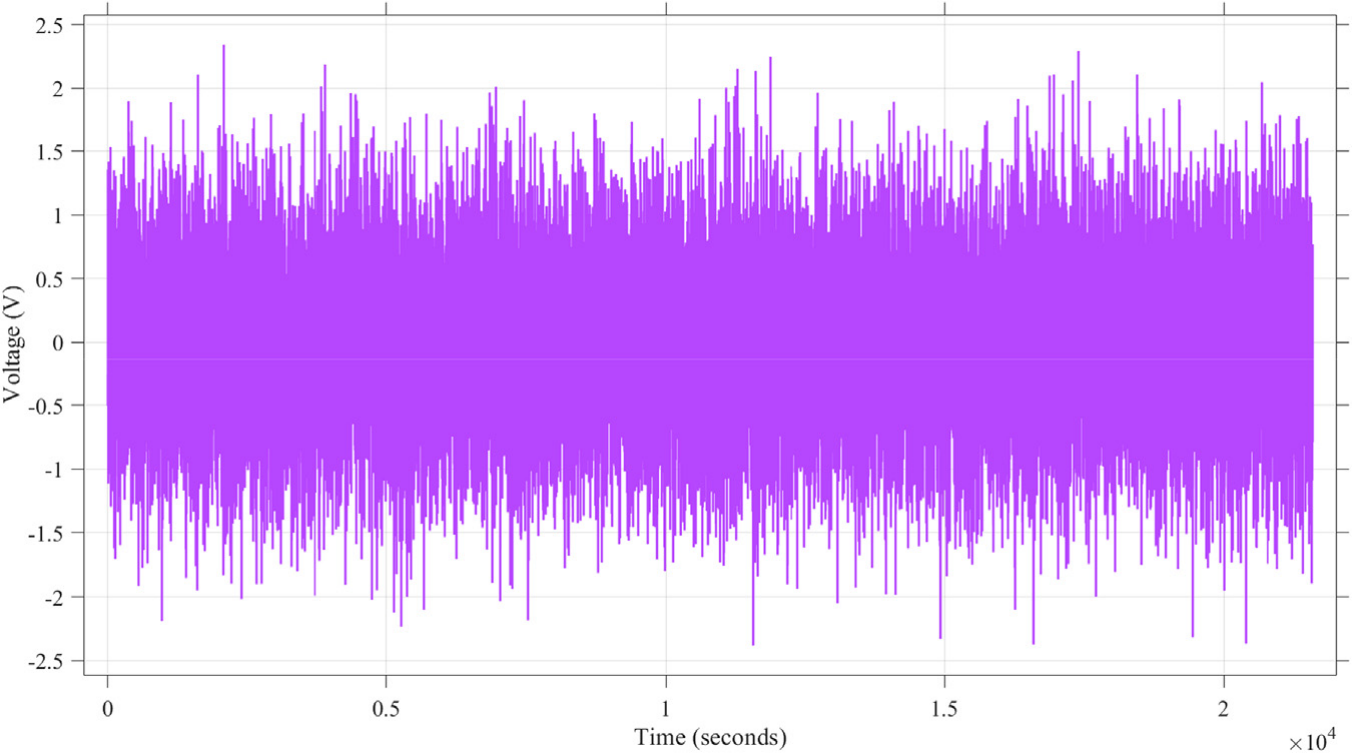
Voltage noise attack using Band-Limited White Noise block noise power of 0.4.

Fig. 13 shows the voltage profile, with noise signal variations ranging between −2.5 V and +2.5 V. This noise disrupts the voltage readings that are crucial for the Kalman Filterʼs state updates. The continuous high-frequency noise is consistent with the adversarial attack strategy, designed to degrade the accuracy of SoC estimation over time.

Together, these figures highlight how adversarial noise signals in current and voltage profiles propagate through the system, resulting in substantial errors in SoC estimation. This demonstrates the critical need for robust detection and mitigation strategies to safeguard against such disruptions.

### 4.6 Inclusion/Exclusion criteria

Inclusion: Only those simulations where the noise signals successfully disrupted the SoC and SoH estimations without exceeding the detection thresholds were included in the dataset.

Exclusion: Scenarios where noise signals were either ineffective or easily detectable were excluded to focus on the most challenging and relevant cases.

### 4.7 Data normalization

The collected data were normalized to standard ranges, ensuring consistency across different simulation runs. Voltage and current data were scaled based on the battery model's operating range, while the SoC and SoH estimates were normalized to percentages.

### 4.8 Code and file

Simulation Code: All code used for setting up the MATLAB/Simscape environment, and running the simulations is included in the supplementary materials. This includes scripts for the battery model, and UKF implementation.

Data Files: The dataset includes .MAT files for each simulation run, containing time-stamped data for voltage, current, SoC, SoH, and noise signals.

## Limitations

One limitation of our work is the absence of a physical battery setup, although we have tested the model using different battery datasheets and estimation algorithms. In our future work, we plan to incorporate a physical battery in a hardware-in-the-loop (HIL) setup, enabling direct integration with our estimation model in Simscape.

## Ethics Statement

The authors have read and follow the ethical requirements for publication in Data in Brief and confirming that the current work does not involve human subjects, animal experiments, or any data collected from social media platforms.

## CRediT Author Statement

**Alaa Selim**: Conceptualization, Methodology, Software, Data curation, Writing, Original draft preparation. **Huadong Mo**: Visualization, Supervision, Investigation, Writing- Reviewing and Editing. **Hemanshu Pota**: Visualization, Supervision, Investigation, Writing- Reviewing and Editing.

## Data Availability

MendeleyDataset of Noise Signals Generated by Smart Attackers for Disrupting State of Health and State of Charge Estimations in Battery Energy Storage Systems (Original data).
